# Dysmenorrhoea

**Published:** 1848-11

**Authors:** 


Dysmenorrhoea.—Dr. H. J. Holmes, of Mississippi, has recently written a letter to the editors of the New Orleans Medical and Surgical Journal, in which he states that the congestive, plethoric or inflammatory form of dysmenorrhœa is easily managed by a well-directed course of treatment. It is only in the irritable form that so much difficulty is encountered, and which has been generally considered incurable. The object of the letter is to throw some light upon the subject of the treatment of this disease, and propose a remedy which never fails to relieve pain, and if continued, will establish the *period*, and make it regular, free and healthy. The frequent trials I have made with it, says Dr. H,, in all the forms of dysmenorrhœa, and particularly in the neuralgic or irritable, has convinced me that it acts specifically upon the uterus, in relieving pain and spasms, increasing the flow, dilating the canal, and permitting coagula or membranes to pass without the least inconvenience. The remedy has been tested fully in a case which I now have uuder treatment. The patient has irritable dysmenorrhœa, accompanied with convulsions, and dependant upon ulceration of the canal and os uteri; the pain has been entirely relieved, but the ulceration still remains, the cure of which I intend to effect by means of the caustic.

The prescription is as follows:

℞. Gm. Camphor, ʒijss.

Ext. Belladonna,

Sulph. Quinine, aa ʒss.

M. ft. et divid. in pil. No. 80.

These pills are to be taken only at each period, and at the commencement of pain. Dose, two every hour until the pain ceases. In extreme cases, they may be given during the intervals of the periods, say one pill two or three times a day.

We have been in constant habit of using the above combination both in public and private practice for the last three years, and know it to be a valuable remedy in irritable dysmenorrhœa. We do not remember of seeing it in print before.—*N. Y. Journal of Med. & Collat. Sciences.*

